# Problematic Use of the Internet in Subjects With Bipolar Disorder: Relationship With Posttraumatic Stress Symptoms

**DOI:** 10.3389/fpsyt.2021.646385

**Published:** 2021-04-26

**Authors:** Claudia Carmassi, Carlo Antonio Bertelloni, Annalisa Cordone, Valerio Dell'Oste, Virginia Pedrinelli, Filippo Maria Barberi, Enrico Massimetti, Eric Bui, Lliliana Dell'Osso

**Affiliations:** ^1^Department of Clinical and Experimental Medicine, University of Pisa, Pisa, Italy; ^2^Caen University Hospital, University of Caen Normandy, Caen, France

**Keywords:** problematic use of the internet, internet addiction, behavioral addiction, bipolar disorder, trauma, PTSD, arousal, TALS-SR

## Abstract

**Background:** Literature shows a high risk for problematic use of the Internet (PUI) in mood disorders, especially in bipolar disorder (BD). In subjects with BD, traumatic events and posttraumatic stress disorder (PTSD) are related to alcohol or substance use disorder, as well as to gambling disorder. However, little is known about the possible association between traumatic exposure and PUI. The present study was aimed at examining the relationship between PUI and trauma exposure, besides PTSD symptoms, in subjects with BD.

**Methods:** A sample of 113 subjects with BD was screened to putative PUI. Furthermore, they completed the Trauma and Loss Spectrum Self-Report (TALS-SR) to assess traumatic events and posttraumatic stress symptoms.

**Results:** Twenty-four subjects (21.2%) reported putative PUI. Subjects with putative PUI presented significantly higher scores in the TALS-SR domains *Potentially Traumatic Events, Re-experiencing, Maladaptive coping*, and *Arousal*, as well as in the TALS-SR total score. In a logistic regression model, a positive association emerged between *Potentially Traumatic Events* and *Arousal* TALS-SR domains and putative PUI.

**Conclusion:** One in five patients with BD screened positive for PUI. A significant association between PUI and lifetime traumatic events as well as PTSD symptoms emerged, highlighting the relevance of the comorbidity between PTSD and PUI in subjects with BD.

## Introduction

Internet use has become common in Western countries and is now present in a substantial part of everyday life (1). The rapid growth of its use during the last decades provided a new environment in which new maladaptive behaviors have progressively emerged (2). It is in fact now recognized the existence of a spectrum of Internet usage, from a controlled and adaptive behavior to an uncontrolled and maladaptive one (3). The expression “Problematic use of the Internet” (PUI) (4) was conceptualized in order to encompass previous different but substantially overlapping conditions, like *Problematic Internet Use, Internet Addiction*, or *Compulsive Internet use* (5–7). It includes a range of “potentially problematic Internet-related behaviors,” including those related to gaming, gambling, buying, pornography viewing, social networking, “cyber-bullying,” and “cyberchondria,” among others (4). PUI was included among the so-called behavioral addictions (8), and it may result in serious psychological, economic, social, school, and working difficulties, leading to functioning impairment (3, 9).

Although growing evidence suggests that PUI is associated with Major Depressive Disorder or depressive symptoms (10–13), the relationship with other mood disorder, particularly bipolar disorder (BD) has been explored to a lesser extent (14). Literature reported an overlap, in neurobiological characteristics, clinical features, and prevalence rates between PUI and BD (15), particularly mania and hypomania. Some authors then proposed to define these two latter conditions as exclusion criteria for Internet-related pathologies, as for other behavioral addictions (16–19). During elevated mood episodes, in fact, increased tendency to PUI may be related to arousal, restlessness, and enhanced excitement or may be a manifestation of an inclination for reward-based behaviors. Conversely, others have suggested that PUI, as well as other behavioral addictions may represent a self-medication strategy for bipolar individuals, to cope with anxiety, tension, and other negative emotions of depressive episodes (20).

Despite other behavioral addictions including gambling disorder, compulsive buying, or sex addiction, are commonly reported in individuals with BD (20–22), scant and still inconclusive data are available on the PUI. On the other hand, some studies reported BD rates among individuals with PUI ranging between 13% and 55% (14, 18, 19). Surveys on adolescents and university students corroborated these previous data on the relationship between PUI and BD (23, 24). However, a recent large study, performed on a cohort of 6,510 Korean subjects, did not find significant differences in the rate of BD in individuals with PUI with respect to those without it (25).

Conversely, to the best of our knowledge, only two studies explored PUI rates in BD patients, reporting similar or lower levels with respect to healthy controls (26, 27). In this regard, it is important to notice that Di Nicola et al. (26) explored PUI rates among patients enrolled until 2008, and Internet usage pattern and PUI conceptualization as an addiction evolved since then; furthermore, Sapir et al. (27) in their study compared a group of bipolar disorder I (BDI) 50 subjects with a healthy control group differing for social or socioeconomic status, pointing out as this variance could influence PUI and other behavioral addiction rates. Thus, the need to examine PUI in bipolar samples remains relevant.

A relationship has been consistently reported between traumatic experiences, posttraumatic stress disorder (PTSD) and addictive behaviors including substance or alcohol use disorders (28–31) and gambling disorder (32). This propensity to addiction in traumatized individuals could represent a maladaptive “self-medication” coping mechanism in order to reduce the hyperarousal component of PTSD (33, 34). Some authors have also suggested that arousal associated with withdrawal may be augmented by PTSD-linked hyperarousal, making the addictive behavior interruption even more difficult (35). The existence of an association between PTSD and gambling is demonstrated by several studies reporting a range of esteemed rates of PTSD between 12.5% and 34% in samples of treatment-seeking gamblers (36–39). It may be hypothesized that the same pattern of association could be observed for other behavioral addictions such as PUI. Most recently, in fact, few but suggestive studies pointed out the relationship between traumatic experiences or stressful life events and PTSD on PUI. Some recent data highlighted an association between a history of emotional or sexual trauma or stressful life events and PUI among adolescent and young adults (40–43). A recent large study conducted on a sample of South Korean students survived to a shipwreck showed that PUI was strongly related to PTSD development (44). Furthermore, posttraumatic stress symptomatology in BD was related to addictive disorders such as alcohol or substance use and, consequently, to a worse outcome (45).

Upon this evidence, we may speculate that a behavioral addiction like PUI may also be related to traumatic exposure and PTSD burden in BD patients, but no data are yet available on this association. The present study aims to explore the impact of posttraumatic stress symptoms on PUI among hospitalized patients with BD.

## Methods

### Participants and Procedures

A consecutive sample of 133 in-patients hospitalized in a major Italian psychiatric clinic aged 18–60 years with a diagnosis of bipolar I or bipolar II disorder were recruited. From the initial sample, *n* = 113 (85%) subjects had usable (non-missing) data for the present study. The enrollment was conducted from November 2016 to December 2018, framed in the “Italian multicenter study for the validation of the Adult Autism Subthreshold Spectrum questionnaire” (Protocol number 551/2015). Inability to understand Italian language or other restrictions in verbal communication was an exclusion criterion. All eligible subjects were informed on study procedures, had opportunity to ask questions, and were asked to provide written informed consent.

### Measures

We used the Structured Clinical Interview for DSM-5 Disorders (SCID-5) (46) to assess BD diagnosis. Furthermore, according to a previous study (5), we utilized the following one-item question to screen putative PUI in the sample: “Do you spend a lot of time playing videogames or surfing on internet, to the extent of forgetting to do routine tasks?.” The item was extrapolated from the Adult Autism Subthreshold Spectrum (AdAS) (47), a questionnaire developed and validated as part of a multicenter study to assess the presence of the wide spectrum of manifestations associated with autism spectrum disorder. Specifically, in the present study, a positive endorsement of the mentioned item indicated putative PUI.

The Trauma and Loss Spectrum Self Report (TALS-SR) is an instrument developed for the assessment of post-traumatic stress symptoms (48–50). It includes 116 items exploring the lifetime experience of a range of losses and/or traumatic events and lifetime symptoms, behaviors, and personal characteristics that might represent manifestations and/or risk factors for the development of a stress-response syndrome. The instrument is organized into nine domains including the following: *loss events* (I); *grief reactions* (II); *potentially traumatic events* (III); *reactions to losses or upsetting events* (IV); *re-experiencing* (V); *avoidance and numbing* (VI); *maladaptive coping* (VII); *arousal* (VIII); and *personal characteristics/risk factors* (IX). The responses to the items are coded in a dichotomous way (yes/no), and domain scores are obtained by counting the number of positive answers. The correlation between the self-report (TALS-SR) and the interview format (SCI-TALS) of the TALS always exceeded the threshold of 0.90, defining a substantial reliability in all the domains (49).

### Statistical Analyses

Chi-square test (or Fisher's test if appropriate) and Student's *t* tests were used to compare sociodemographic and clinical characteristics, as well as TALS-SR domains between individuals with and without PUI. Furthermore, a logistic regression analysis was performed to examine the association of TALS-SR domains (as independent variables) with the presence of putative PUI (as the dependent variable), after controlling for the effects of potentially confounding variables age and gender. In order to examine whether *arousal* mediated the association between traumatic exposure and PUI, two logistic regression models were conducted, as follows: the first for subjects with low arousal symptoms (TALS-SR *arousal* domain under the median of the total sample 2); the second for subjects with high arousal symptoms (TALS-SR *arousal* domain over the median of the total sample 2). Chi-square test (or Fisher's test if appropriate) was computed to compare the endorsement rates of the items of the TALS-SR *arousal* domain (VIII) between subjects with PUI and without ones. The statistical analyses were carried out using SPSS version 25.0. The level of statistical significance was set to *p* < 0.05 (two-tailed).

## Results

In the study sample, the mean age was 43.0 ± 12.5 years, and 72 participants (63.7%) were males. Twenty-seven subjects (25.5%) had college degree, the majority was employed (*n* = 64, 59.8%), and 42 (39.6%) subjects were married. Furthermore, 85 (80.2%) patients had a positive family psychiatric history, bipolar disorder I (BDI) was diagnosed in 73 (67.0%) patients while bipolar disorder II (BDII) in 36 of them (33.0%). Participants were almost equally divided in depressive (55, 48.7%) and manic/hypomanic episode polarity at the time of the hospital admission (58, 51.3%).

In the total sample, 24 (21.2%) subjects reported putative PUI. No significant differences emerged in sociodemographic and clinical characteristic between BD subjects with and without PUI, except for age which was about 10 years lower among the former (34.46 ± 12.93 vs. 44.93 ± 11.57, *p* < 0.001) (see Table 1).

**Table 1:** Sociodemographic and clinical characteristics in BD subjects with (*N* = 24) and without PUI ones (*N* = 89).

	**PUI**	**No-PUI**	***p***
	***N*** **(%)**	***N*** **(%)**	
Gender			
Male	19 (79.2)	53 (59.6)	0.125
Female	5 (20.8)	36 (40.4)	
Education			
Not graduated	18 (85.7)	61 (71.8)	0.301
Graduated	3 (14.3)	24 (28.2)	
Missing data	3	4	
Occupation			
Unemployed	12 (54.5)	31 (36.5)	0.194
Employed	10 (45.5)	54 (63.5)	
Missing data	2	4	
Marital status			
Single/divorced/widowed	15 (71.4)	49 (57.7)	0.364
Married	6 (28.6)	36 (42.3)	
Missing data	3	4	
Psychiatric family history			
Negative	7 (33.3)	14 (16.5)	0.080
Positive	14 (66.7)	71 (83.5)	
Missing data	3	4	
Type of bipolar disorder			
BD-I	18 (75.0)	55 (64.7)	0.245
BD-II	6 (25.0)	30 (35.3)	
Missing data		4	
Current episode polarity			
Depressive	9 (37.5)	46 (51.7)	0.158
Manic/Hypomanic	15 (62.5)	43 (48.3)	
	Mean ± SD	Mean ± SD	*p*
Age (years)	34.46 ± 12.93	45.03 ± 11.59	<0.001

Subjects with PUI reported significantly higher mean scores in the TALS-SR domains *potentially traumatic events* (III), *Re-experiencing* (IV), *Maladaptive coping* (VII), and *Arousal* (VIII) and the TALS-SR total score, compared with no-PUI ones (Table 2).

**Table 2:** Comparison of TALS-SR domains and total score between BD subjects with (*N* = 24) and without PUI (*N* = 89).

	**PUI**	**No-PUI**	***p***
	**(mean ± SD)**	**(mean ± SD)**	
(I) Loss events	4.83 ± 2.01	4.84 ± 2.09	0.807
(II) Grief reactions	15.67 ± 5.61	12.90 ± 5.96	0.072
(III) Potentially traumatic events	8.21 ± 5.31	4.86 ± 3.26	0.004
(IV) Reactions to losses or upsetting events	9.25 ± 4.19	8.17 ± 4.06	0.312
(V) Re-experiencing	5.17 ± 2.65	4.02 ± 2.29	0.042
(VI) Avoidance and numbing	6.45 ± 2.75	4.88 ± 2.99	0.024
(VII) Maladaptive coping	3.12 ± 2.49	2.02 ± 1.90	0.050
(VIII) Arousal	3.33 ± 1.63	2.25 ± 1.62	0.006
(IX) Personal characteristics/risk factors	2.50 ± 1.53	2.61 ± 1.65	0.957
Total TALS-SR score	58.54 ± 22.32	46.57 ± 17.63	0.034

In the logistic regression on the total sample, the TALS-SR Domains *Potentially traumatic events* (III) [*b* = 0.21 (SE = 0.097), *p* = 0.027], *Arousal* (VIII) [*b* = 0.58 (SE = 0.264), *p* = 0.027], and *Personal characteristics/risk factors* (IX) [*b* = −0.51 (SE = 0.228), *p* = 0.024] were associated with the PUI (see Table 3).

**Table 3:** Logistic regression analysis: age, gender, and TALS-SR domains as predictive factors associated with PUI in the total sample (*N* = 113).

**Predictive factors**	***B* (S.E.)**	**O.R**.	**95% CI**	***p***
(I) Loss events	−0.31 (0.198)	0.73	0.50–1.08	0.117
(II) Grief reactions	0.12 (0.069)	1.13	0.98–1.29	0.082
(III) Potentially traumatic events	0.21 (0.097)	1.24	1.02–1.50	0.027
(IV) Reactions to losses or upsetting events	−0.05 (0.110)	0.94	0.76–1.17	0.604
(V) Re-experiencing	0.04 (0.181)	1.04	0.73–1.49	0.811
(VI) Avoidance and numbing	0.04 (0.143)	1.04	0.79–1.34	0.780
(VII) Maladaptive coping	−0.23 (0.210)	0.80	0.53–1.20	0.273
(VIII) Arousal	0.58 (0.264)	1.80	1.07–3.00	0.027
(IX) Personal characteristics/risk factors	−0.51 (0.228)	0.60	0.38–0.93	0.024
K	1.15(1.540)	3.14	–	0.457

*Cox R^2^ = 0.286; Nagelkerke R^2^ = 0.444*.

*Hosmer-Lemeshow test: χ^2^ = 6.843, p = 0.554*.

*Global-goodness-fit percentage = 83.2%*.

We conducted two logistic regression analyses to assess the possible relationship between the TALS-SR Domain *exposure to traumatic event* (III) as an independent variable and the putative PUI as the dependent variable. The first regression was performed in the subjects with low arousal symptoms and was not significant [*b* = −0.18 (SE = 0.14), *p* = 0.982] (see Table 4A). Conversely, the regression conducted in subjects with high arousal symptoms, showed a significant association between *exposure to traumatic event* (III) and PUI [*b* = 0.27 (SE = 0.09), *p* = 0.002] (see Table 4B). This result pointed out a possible mediation effect of arousal on the association between traumatic exposure and PUI.

**Table 4A:** Logistic regression analysis: TALS-SR Domain III (*potentially traumatic events*) as predictive factor associated with PUI in BD patients with low arousal symptoms.

**Predictive factors**	***B* (S.E.)**	**O.R**.	**95% CI**	***p***
(III) Potentially traumatic events	−0.18 (0.14)	0.98	0.75–1.28	0.982

*Cox R^2^ <0.001; Nagelkerke R^2^ = 0.001*.

*Hosmer-Lemeshow test: χ^2^ = 3.664, p = 0.722*.

*Global-goodness-fit percentage = 85.7%*.

**Table 4B:** Logistic regression analysis: TALS-SR Domain III (*potentially traumatic events*) as predictive factor associated with PUI in BD patients with high arousal symptoms.

**Predictive factors**	***B* (S.E.)**	**O.R**.	**95% CI**	***p***
(III) Potentially traumatic events	0.274 (0.09)	1.31	1.10–1.56	0.002

*Cox R^2^ = 0.201; Nagelkerke R^2^ = 0.290*.

*Hosmer-Lemeshow test: χ^2^ = 7.261, p = 0.402*.

*Global-goodness-fit percentage = 77.2%*.

Finally, subjects with PUI presented a significantly higher endorsement rates of TALS-SR arousal domain items *n* = 105 (…* have trouble concentrating or paying attention, for example, following the story line of a TV program or book or remembering what you had read?)* and *n* = 106 (…* feel like you just couldn't relax or let your guard down?*) (see Table 5).

**Table 5:** TALS-SR *arousal* domain (VIII) item endorsement rates between BD subjects with (*N* = 24) and without PUI ones (*N* = 89).

	**PUI**	**No-PUI**	***p***
	***N* (%)**	***N* (%)**	
105)…* have trouble concentrating or paying attention?*	21 (87.5%)	54 (60.7%)	0.026
106)…* feel like you just couldn't relax or let your guard down?*	19 (79.25%)	42 (47.2%)	0.011
107)…* startle easily at the sound of sudden noises, or when someone touched you, spoke to you, or approached you unexpectedly?*	14 (58.3%)	35 (39.3%)	0.151
108)…* feel more irritable, have outbursts of anger or rage, or lose your temper over minor things?*	14 (58.3%)	37 (41.6%)	0.217
109)…* have more difficulty falling asleep or staying asleep than before or need a light on to go to sleep?*	12 (50.0%)	33 (37.1%)	0.361

## Discussion

To the best of our knowledge, this is the first study exploring the associations between PUI and posttraumatic stress symptoms among subjects with BD. In particular, we found putative PUI in one in five subjects. Individuals with BD and PUI reported significantly more lifetime traumatic events with respect to no-PUI ones, as well as higher levels of posttraumatic stress symptoms.

The rates of PUI reported in the present study are in line with previous studies describing high rates of both drug-related and behavioral addictions in BD (20), despite appearing to be slightly higher than those specifically reporting PUI rates. Methodological differences may render comparison difficult (26, 27) corroborating the fact that the relationship between PUI and mood disorders has resulted to be complex (15) and the comorbidity rates are still object of arguing. However, our results corroborate a recent large meta-analysis concluded underlining the need to undertake systematic and routine screening and comprehensive assessment of possible co-occurring behavioral addictions, including PUI, among patients with BD. In particular, the authors addressed the potential negative impact that these disorders may have directly on the bipolar illness and, more in general, to the impairment in the social, relational, and economic quality of life (20). In light of these evidence, exploring the determinant of these comorbidity appears to be a critical issue for researchers, in order to develop specific prevention or treatment strategies.

The present study first shed light on the role of traumatic events and the prevalence of PTSD symptoms in BD patients. This is a particularly promising research pathway, as suggested by the results of the few studies that have been focusing on PUI in PTSD (44). Literature, in fact, showed a relationship between a history of stressful events or sexual abuse and PUI among adolescents and young adults (40–43) and an increased risk of PTSD development among adolescents with PUI (25). While research on this issue is to date at its preliminary stage, the association between PTSD and other addiction disorders such as substance or alcohol use disorder and gambling disorder, are widely acknowledged (32, 51, 52). Furthermore, several studies highlighted how PTSD negatively affects the course of BD (53–57) and increases the risk of drug-related addictions (45, 58). We add to the literature that also PUI, as alcohol, substance, and gambling, is related to posttraumatic stress symptoms in bipolar patients. These data also suggest a detrimental role for PTSD hyperarousal symptoms, especially attention deficit and startle, on PUI like that previously reported for alcohol or substance use. In this regard, some authors reported how hyperarousal is the most prominent symptom cluster in determining PTSD severity over time (59). Furthermore, in several studies, hyperarousal symptoms were specifically associated with substance abuse among subjects with PTSD (60–63). Recently, Green et al. showed a relationship between arousal levels and gambling disorder, suggesting a role for hyperarousal in non-substance-related addictive disorders (34). The results of the present study pointed out the relevant role of arousal in bipolar patients with PUI, corroborating previous studies and suggesting that these symptoms may have a significant role in PUI comorbidity too. Interestingly, risk factors for PTSD, such as impulsivity or being sensitive to loss, were negatively related to PUI. It is in contrast with previous studies showing an association between impulsivity and PUI (64, 65). This difference may be due to our sample characteristics. We may speculate that in traumatized subjects with BD, these personal features could be related to other kinds of maladaptive behaviors or other addictions.

In our study, the temporal relationship between PUI and posttraumatic stress symptoms remains unclear. On one hand, we may speculate that bipolar patients who experienced a traumatic event tend to use the Internet excessively in order to cope with negative emotions and anxious states linked to posttraumatic stress symptoms. On the other hand, subjects reporting PUI may be more vulnerable to stressful events or more prone to develop PTSD. Given that a causal link cannot be obtained by our results, previous studies, in accordance with the former hypothesis, showed an association between childhood trauma experience and PUI, highlighting the possible role of PUI as a maladaptive coping method to traumatic events and distressful memories (40, 43).

Despite our interesting results, some limitations should be acknowledged. The most important one is the assessment of PUI using an item from the AdAS Spectrum questionnaire that may not be specific enough, and future studies will need to use diagnostic interviews. A second limitation is the relatively small sample size. The third limitation is the lack of assessment of other psychiatric comorbidities, such as anxiety, which might have been potential confounds. Another limitation is the cross-sectional design of the study and the lack of information on the onset and the duration of the PUI and the PTSD. Furthermore, the assessments were performed during an acute phase of the BD, and this may have affected the results. Finally, PTSD symptoms were assessed with a self-report scale. However, the self-report version of the TALS had previously demonstrated good psychometric properties and a high correlation with the clinician-rated version.

In conclusion, we found that putative PUI is quite common in subjects with BD and is associated with history of traumatic events as well as posttraumatic stress symptoms. Particularly, in a logistic regression model, hyperarousal and a history of potentially traumatic events predicted the presence of putative PUI, highlighting their role in patients with PUI. We may argue that in light of these results, systematical use of trauma-focused therapeutic approaches, such as cognitive behavioral therapy or prolonged exposure therapy, should be even more encouraged. These psychotherapies, in fact, may not only improve patient's posttraumatic stress symptoms in mood disorders (66–68) but also reduce the risk of developing behavioral addiction disorders such as PUI. Future longitudinal research is warranted to better understand the link between posttraumatic stress symptoms and PUI in BD. This could lead to an improvement in the development of tailored assessment and interventions of patients with BD.

## Data Availability Statement

The datasets generated for this article are not readily available because, the data supporting the findings of the article are not publicly available, but it can be provided by the corresponding author on reasonable request. Requests to access the datasets should be directed to Dr. Carlo Antonio Bertelloni carlo.ab@hotmail.it.

## Ethics Statement

The studies involving human participants were reviewed and approved by Comitato Etico Regionale per la Sperimentazione Clinica della Regione Toscana AREA VASTA NORD OVEST (CEAVNO, Pisa, Italy). The patients/participants provided their written informed consent to participate in this study.

## Author Contributions

All authors gave substantial contribution to the study and approved the final version of the manuscript and the manuscript submission to Frontiers in Psychiatry.

## Conflict of Interest

The authors declare that the research was conducted in the absence of any commercial or financial relationships that could be construed as a potential conflict of interest.
